# Hypoxia and Activation of Neutrophil Degranulation-Related Genes in the Peripheral Blood of COVID-19 Patients

**DOI:** 10.3390/v16020201

**Published:** 2024-01-28

**Authors:** Hongxing Lei

**Affiliations:** 1 CAS Key Laboratory of Genome Sciences and Information, Beijing Institute of Genomics, Chinese Academy of Sciences, and China National Center for Bioinformation, Beijing 100101, China; leihx@big.ac.cn; Tel.: +86-010-84097276; 2 Cunji Medical School, University of Chinese Academy of Sciences, Beijing 101408, China; 3 Center of Alzheimer’s Disease, Beijing Institute for Brain Disorders, Beijing 100069, China

**Keywords:** COVID-19, neutrophil degranulation, hypoxia, pandemic, respiratory viral infection

## Abstract

Severe COVID-19 is characterized by systematic hyper-inflammation and subsequent damage to various organs. Therefore, it is critical to trace this cascade of hyper-inflammation. Blood transcriptome has been routinely utilized in the interrogation of host immune response in COVID-19 and other infectious conditions. In this study, consensus gene dysregulation in the blood was obtained from 13 independent transcriptome studies on COVID-19. Among the up-regulated genes, the most prominent functional categories were neutrophil degranulation and cell cycle, which is clearly different from the classical activation of interferon signaling pathway in seasonal flu. As for the potential upstream causal factors of the atypical gene dysregulation, systemic hypoxia was further examined because it is much more widely reported in COVID-19 than that in seasonal flu. It was found that both physiological and pathological hypoxia can induce activation of neutrophil degranulation-related genes in the blood. Furthermore, COVID-19 patients with different requirement for oxygen intervention showed distinctive levels of gene expression related to neutrophil degranulation in the whole blood, which was validated in isolated neutrophils. Thus, activation of neutrophil degranulation-related genes in the blood of COVID-19 could be partially attributed to hypoxia. Interestingly, similar pattern was also observed in H1N1 infection (the cause of Spanish flu) and several other severe respiratory viral infections. As for the molecular mechanism, both HIF-dependent and HIF-independent pathways have been examined. Since the activation of neutrophil degranulation-related genes is highly correlated with disease severity in COVID-19, early detection of hypoxia and active intervention may prevent further activation of neutrophil degranulation-related genes and other harmful downstream hyper-inflammation. This common mechanism is applicable to current and future pandemic as well as the severe form of common respiratory infection.

## 1. Introduction

While the World Health Organization (WHO) chief declared the end of the global health emergency for COVID-19 in 2023 (https://www.who.int/, accessed on 15 May 2023), numerous questions about this pandemic disease persist. Crucially, how can we improve the treatment of this respiratory infection? Despite the great effort in the innovative development of vaccines and drugs [1,2], too many lives have been lost in this pandemic. For a disease dominated with problems in immune dysregulation [3], we may have to dig deeper in our understanding of the cascade of host immune response to respiratory viral infection.

Host immune response to viral or bacterial infection is a double-edged sword. We rely on a swift immune response to eliminate the invading pathogens. However, sustained immune response may go awry, leading to hyper-inflammation and systemic damage to our own body. “Cytokine storm” has been implicated as a leading cause of death for COVID-19 [4]. Unfortunately, when a “cytokine storm” is observed, it may be too late to act upon. Thus, it is crucial to gain novel insight into host response in the early course of the disease and discover early actionable warning signs.

Blood transcriptome has been widely used to examine the host response to infection including COVID-19. In our previous works, we noticed atypical immune dysregulation in COVID-19 using a few prominent marker genes for host response to viral or bacterial infection [5,6]. To reach a more comprehensive view of host response in COVID-19, transcriptome-wide gene expression change was examined in this work based on over a dozen of independent datasets related to hospitalized COVID-19 patients. Consensus gene expression change was obtained, and functional enrichment of the consensus genes was revealed. 

To go one step further, it will be valuable to find out what may have contributed to the atypical immune gene dysregulation in the early stage of COVID-19. Unlike those in mild respiratory infections such as seasonal flu, hypoxia has been observed in a significant portion of COVID-19 patients and implicated with disease severity [7]. Some patients with unnoticed hypoxia in the early stage developed severe symptoms later [8]. Thus, hypoxia is a candidate of potential causal factors for the atypical immune gene dysregulation in COVID-19. Investigation along this line was the main focus of the current work. The clinical implications were discussed subsequently.

## 2. Materials and Methods

### 2.1. Analysis of Peripheral Gene Dysregulation in COVID-19

To obtain consensus gene dysregulation in COVID-19, 13 transcriptome datasets were collected from public resources. For consistency, the tissue source was restricted to whole blood across all 13 datasets. The selection criteria also required the inclusion of both hospitalized COVID-19 patients and healthy controls in every dataset. Among the 13 datasets, 11 datasets were downloaded from gene expression omnibus (GEO, https://www.ncbi.nlm.nih.gov/geo, accessed on 15 May 2023), 1 dataset was downloaded from the Zenodo repository (https://zenodo.org/, accessed on 15 May 2023), and 1 more was downloaded from the genome sequence archive (GSA, https://ngdc.cncb.ac.cn/gsa/, accessed on 15 May 2023). The accession numbers were GSE152641 [9], GSE161731 [10], GSE161777 [11], GSE171110 [12], GSE179850 [13], GSE185557 [14], GSE189990 [15], GSE196822 [16], GSE213313 [17], GSE217948 [18], GSE223885 [19], Zenodo 6120249 [20], and HRA000238 [21]. 

A total of 11 of the 13 transcriptome studies on COVID-19 employed RNA-Seq (RNA sequencing) technology developed at Illumina. One study (GSE223885) employed nanopore technology (a single-molecule sequencing technology) developed at Oxford Nanopore and another study (GSE213313) utilized microarray technology developed at Agilent. In these studies, all of the COVID-19 patients tested positive for SARS-CoV-2 infection and had symptoms compatible with COVID-19 infection at the time of hospital admission. In total, 1087 samples were included in the examination of consensus gene dysregulation in COVID-19.

Differentially expressed genes (DEGs) were calculated by DESeq2 for RNA-Seq datasets with raw counts available [22]. It required two pre-compiled files, one for the gene expression matrix (raw counts) and another for the sample information (case or control status). The calculation was done in R (https://www.r-project.org/, accessed on 15 May 2023) and required the pre-loading of the DESeq2 package. For datasets GSE189990, GSE213313, GSE217948, GSE223885 and HRA000238, DEG lists were directly obtained from the original publications. To reduce noise in high-throughput data, only genes with fold change > 2 or <0.5 and adjusted *p* value < 0.05 in the case-control comparison were selected for further analysis. Genes that occurred in at least 8 of the 13 DEG lists (>60%) were retained as the consensus DEGs. Functional enrichment analysis was conducted on consensus DEGs using the ToppFun module on the ToppGene web server (https://toppgene.cchmc.org/, accessed on 15 May 2023). It required the input of a gene list. To avoid large functional groups with broad function, the group size was limited to within 1000 genes for the enrichment analysis. From the output of the enrichment analysis, we focused on the “Pathway” enrichment, which is most relevant to the clearly defined cellular function. Among the most consensus DEGs (in at least 12 of the 13 DEG lists), 13 genes within the functional category of neutrophil degranulation (NDG) were selected for more in-depth downstream analysis.

### 2.2. Analysis of the Link between Hypoxia and Gene Dysregulation

The effect of systemic hypoxia on gene dysregulation was evaluated using four transcriptome datasets, including two RNA-Seq datasets for physiological hypoxia (high altitude-related, GSE164890 and GSE196728) [23,24] and two microarray datasets for pathological hypoxia (stroke-related, GSE36791 and GSE58294) [25,26]. The RNA-Seq datasets were downloaded directly from the corresponding GEO web pages. The raw gene counts were normalized and log2 transformed for downstream analysis. The microarray datasets were downloaded using a simple R script, which included the mapping of the probe IDs to genes. The expression values of the representative NDG genes were extracted from these datasets. The average expression value of the 13 representative NDG genes was used as the NDG gene expression value. For the dataset GSE196728, the participants were sampled at multiple time points upon arrival at the specific locations. A similar trend was observed at different time points, so only one time point (22 h post arrival) was selected for presentation. For these four datasets, the group comparison was done in R using *t*-test (paired *t*-test for same people comparison and unpaired *t*-test for case/control comparison). The figures were drawn with the ggplot2 package in R. 

The correlation between NDG gene expression and oxygen requirement in COVID-19 patients was evaluated using four RNA-Seq transcriptome datasets, including two datasets on whole blood (Zenodo 6120249 and GSE157103) [20,27] and two datasets on isolated neutrophils (GSE212041 and EGAS00001004503) [28,29]. The GEO datasets were downloaded directly from the corresponding GEO web pages. The Zenodo dataset was downloaded from the Zenodo repository (https://zenodo.org/, accessed on 15 May 2023). The EGA dataset was downloaded from the European Genome-phenome archive (https://ega-archive.org/, accessed on 15 May 2023). The raw gene counts were normalized and log2 transformed for downstream analysis. For these four datasets, multi-group comparison was conducted in R using one-way ANOVA followed by Tukey’s test (Tukey’s Honest Significant Differences).

### 2.3. Analysis of Gene Dysregulation in Other Severe Respiratory Infection

Activation of neutrophil degranulation-related genes in other severe respiratory infection was evaluated using five transcriptome datasets, including microarray datasets GSE111368 for H1N1 infection [30], GSE114466 for H7N9 infection [31], GSE101702 for severe influenza A infection [32], and GSE77087 for RSV infection [33], and RNA-Seq dataset GSE196399 for severe community acquired pneumonia [34]. The processing and analysis of these five datasets were similar to the four COVID-19 datasets with different requirement of oxygen intervention mentioned in the preceding paragraph. For these five datasets, multi-group comparison was conducted in R using one-way ANOVA followed by Tukey’s test, while two-group comparison was conducted in R using the unpaired *t*-test.

### 2.4. Analysis of Hypoxia Sensing Pathways and Neutrophil Degranulation

For all of the 13 datasets in the 2 preceding sections, the hypoxia sensing pathways were examined using 2 representative genes: HIF1A for HIF-dependent pathway and PIK3CG for HIF-independent pathway [35]. The normalized expression values for HIF1A and PIK3CG were extracted for each dataset, and the correlation between HIF1A or PIK3CG expression and NDG expression was calculated for each dataset. The plots were drawn in R using ggplot2.

## 3. Results

### 3.1. Activation of Neutrophil Degranulation-Related Genes in the Blood of COVID-19 Patients

To obtain consensus gene dysregulation in COVID-19 patients, 13 independent transcriptome datasets on whole blood were examined (Table 1). In total, 1087 samples were included in the analysis (874 cases and 213 controls). For each dataset, group comparison was conducted between hospitalized COVID-19 patients and healthy controls. To ensure robustness, only genes with fold change above 2 in either direction (plus adjusted *p*-value < 0.05) were considered as differentially expressed genes (DEGs). Consensus DEGs were defined as DEGs in at least 8 of the 13 datasets (>60%), further ensuring robustness. In total, there were 287 consensus DEGs for COVID-19 (whole blood), including 263 up-regulated genes and 24 down-regulated genes (Supplementary Table S1). 

Functional enrichment analysis on the consensus DEGs showed that the up-regulated genes were mainly enriched in neutrophil degranulation (*p* = 2.11 × 10^−35^) and cell cycle (*p* = 5.37 × 10^−13^) (Supplementary Table S2). There are 478 curated genes in the neutrophil degranulation pathway. Among these, 60 genes were found in the list of 263 consensus up-regulated genes (22.8%). No significant functional enrichment was observed for the down-regulated genes, partially due to the small number of genes (only 24 genes). Among the 16 most consensus genes (DEGs in at least 12 of the 13 datasets), 13 belonged to the functional category of neutrophil degranulation (Table 2), including *ARG1*, *CEACAM8*, *RNASE2*, *CD177*, *CKAP4*, *ELANE*, *HP*, *MCEMP1*, *MMP9*, *MPO*, *S100A12*, *S100A9*, and *TCN1*. Thus, neutrophil degranulation will be the main focus of the current work. The median log transformed fold changes were between 1.43 and 4.98 for these 13 genes. It is interesting that these 13 genes all displayed moderate to strong correlation with disease severity in a related COVID-19 study (0.61 < r < 0.77, Table 2) [18]. These 13 genes will serve as surrogates to evaluate the activation of neutrophil degranulation (NDG)-related genes in COVID-19 and other conditions in this study.

### 3.2. Neutrophil Degranulation-Related Genes Can Be Activated by Systemic Hypoxia

Previous works have shown that the classical host response to respiratory viral infection is the activation of interferon-stimulated genes (ISGs), while the activation neutrophil degranulation-related genes is a hallmark of host response to bacterial infection. Thus, it is interesting to find out what may have contributed to the atypical host response in COVID-19. Notably, systemic hypoxia has been observed in a significant portion of hospitalized COVID-19 patients and implicated with disease severity, also an atypical feature for respiratory viral infection such as seasonal flu. As indicated in Table 2, NDG gene expression is also highly correlated with disease severity in COVID-19. Therefore, the potential link between systemic hypoxia and gene expression related to neutrophil degranulation was investigated in this study (Table 3).

**Figure 1:**
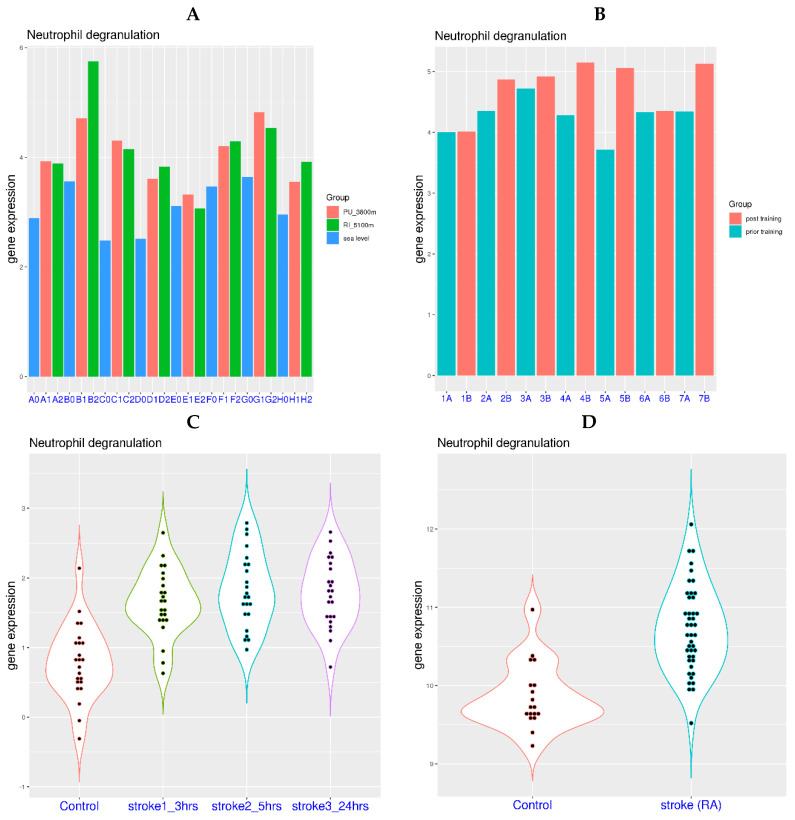
Activation of neutrophil degranulation-related genes in blood by physiological and pathological hypoxia. **Upper left** (**A**): Activation of neutrophil degranulation-related genes at high altitude (red color for 3800 m and green color for 5100 m) compared to sea level (blue color). Eight healthy subjects (marked as A to H) were examined at three locations (dataset GSE196728). **Upper right** (**B**): Activation of neutrophil degranulation-related genes after high intensity training at high altitude (cyan color for before exercise and red color for after exercise). Seven elite athletes (marked as 1 to 7) were examined at 1850 m above sea level (dataset GSE164890). **Lower left** (**C**): Activation of neutrophil degranulation-related genes at 3 time points (3, 5 and 24 h post stroke onset) after cardioembolic stroke (dataset GSE58294). **Lower right** (**D**): Activation of neutrophil degranulation-related genes after ruptured intracranial aneurysm (dataset GSE36791).

Traveling to high altitude can lead to physiological hypoxia for those dwelling at sea level. In a related study, gene expression of the blood was examined for eight healthy participants at both sea level and high altitude (3800 m and 5100 m) [24]. It was evident that NDG gene expression was generally higher at high altitude compared to that at sea level (Figure 1A). The difference of NDG gene expression was 1.04, 1.15, 1.83, 1.10, 0.21, 0.75, 1.19, and 0.59 for the 8 subjects at 3800 m (mean difference 0.98), and it was 1.00, 2.18, 1.67, 1.32, −0.03, 0.82, 0.90, and 0.96 at 5100 m (mean difference 1.10). Using NDG gene expression at sea level as the reference, the *p*-values from paired *t*-tests were 0.0007 and 0.002 for NDG gene expression at 3800 m and 5100 m, respectively. 

Additionally, a session of intense sports training at high altitude may also lead to temporary systemic hypoxia. In a related study, gene expression of peripheral blood was investigated for 7 elite skaters before and after a training session at moderately high altitude (1850 m) [23]. It was evident that NDG gene expression was generally elevated post training (Figure 1B). The difference of NDG gene expression was 0.01, 0.51, 0.20, 0.88, 1.35, 0.02 and 0.78 for the 7 subjects post training compared to those prior training (mean difference 0.54). Using NDG gene expression prior to training as the reference, the *p*-values from paired *t*-tests was 0.029 for NDG gene expression post training. Based on the observations from these two studies, physiological hypoxia can be a causal factor for the elevation of NDG gene expression in blood. 

In addition to physiological hypoxia, certain pathological conditions may also lead to systemic hypoxia due to problems with blood flow. In a study with cardioembolic stroke (Figure 1C) [26], it was evident that NDG gene expression was significantly elevated after the stroke event. The mean NDG gene expression was 0.79 for the control group, while it was 1.64, 1.82, and 1.78 for patients 3 h, 5 h, and 24 h post stroke event, respectively. Using NDG gene expression of the control group as the reference, the *p*-values from *t*-tests were 9.85 × 10^−7^, 5.28 × 10^−8^, and 4.80 × 10^−8^ for NDG gene expression in patients 3 h, 5 h, and 24 h post stroke event, respectively. 

In another study with ruptured intracranial aneurysm (Figure 1D) [25], it was also clear that NDG gene expression was significantly elevated post stroke event. The mean NDG gene expression was 9.86 for the control group, while it was 10.72 for patients post stroke event. Using NDG gene expression of the control group as the reference, the *p*-value from *t*-test was 5.56 × 10^−8^ for NDG gene expression in patients post stroke event. Based on these two studies related to stroke, acute pathological hypoxia can also be a causal factor for the activation of neutrophil degranulation-related genes in blood. Thus, neutrophil degranulation-related genes in blood can be activated by both physiological and pathological hypoxia. 

### 3.3. Oxygen Requirement and Gene Expression Related to Neutrophil Degranulation in COVID-19

**Figure 2:**
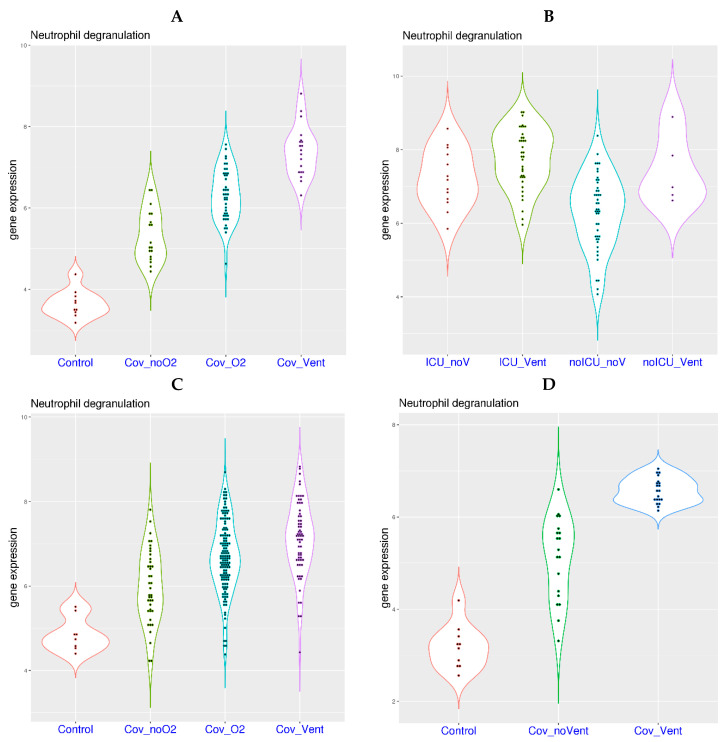
Enhanced activation of neutrophil degranulation-related genes with increased level of oxygen requirement in COVID-19 patients (both whole blood and isolated neutrophils). **Upper left** (**A**): Activation of neutrophil degranulation-related genes in hospitalized COVID-19 patients with room air (Cov_noO2), oxygen supplementation (Cov_O2) and mechanical ventilation (Cov_Vent, dataset Zenodo 6120249, whole blood). **Upper right** (**B**): Activation of neutrophil degranulation-related genes in four groups of hospitalized COVID-19 patients divided by the requirement of ventilation and ICU care (dataset GSE157103, whole blood). **Lower left** (**C**): Activation of neutrophil degranulation-related genes in hospitalized COVID-19 patients with room air (Cov_noO2), oxygen supplementation (Cov_O2) and mechanical ventilation (Cov_Vent, dataset GSE212041 on neutrophils). **Lower right** (**D**): Activation of neutrophil degranulation-related genes in hospitalized COVID-19 patients with or without the requirement of mechanical ventilation (dataset EGAS00001004503 on neutrophils).

If systemic hypoxia is indeed partially responsible for the activation of neutrophil degranulation-related genes in COVID-19, it is expected that further activation of neutrophil degranulation-related genes will be observed in COVID-19 patients with oxygen requirement. Indeed, higher level of NDG gene expression was observed in the blood of COVID-19 patients with higher level of oxygen requirement in a related study (Figure 2A) [20]. More specifically, increasing NDG gene expression was observed in the four sample groups, including healthy controls (mean value 3.65), COVID-19 patients on room air (Cov_noO2, mean value 5.32), patients on supplementary oxygen (Cov_O2, mean value 6.32), and patients on mechanical ventilation (Cov_Vent, mean value 7.40). The *p*-values from multi-group Tukey’s test were 0 for Cov_noO2 compared with controls, 7.0 × 10^−7^ for Cov_O2 compared with Cov_noO2, and 1.0 × 10^−7^ for Cov_Vent compared with Cov_O2.

In another study on whole blood [27], COVID-19 patients were divided into ICU and non-ICU groups, both including patients on ventilation or not on ventilation. Compared to non-ICU patients not on ventilation (noICU_noV, mean value 6.31), higher NDG gene expression was observed in the other three patient groups (Figure 2B), including non-ICU patients on ventilation (noICU_Vent, mean value 7.42), ICU patients not on ventilation (ICU_noV, mean value 7.22), and ICU patients on ventilation (ICU_Vent, mean value 7.66). The *p*-values from multi-group Tukey’s test were 0.05 for comparisons of noICU_noV versus noICU_Vent, 0.01 for noICU_noV versus ICU_noV, and 0 for noICU_noV versus ICU_Vent.

Compared to using whole blood as the tissue source, neutrophil degranulation signal can be more clearly characterized in isolated neutrophils. Consistent with observations from whole blood, a higher level of NDG gene expression was observed in COVID-19 patients with a higher level of oxygen requirement in a study on isolated neutrophils (Figure 2C) [28]. More specifically, increasing NDG gene expression was observed in the four sample groups, including healthy controls (mean value 4.86), COVID-19 patients on room air (Cov_noO2, mean value 5.98), patients on supplementary oxygen (Cov_O2, mean value 6.69), and patients on mechanical ventilation (Cov_Vent, mean value 7.14). The *p*-values from multi-group Tukey’s test were 0.004 for Cov_noO2 compared with controls, 5.0e-05 for Cov_O2 compared with Cov_noO2, and 0.006 for Cov_Vent compared with Cov_O2.

In another study on isolated neutrophils [29], a similar trend was observed (Figure 2D). More specifically, increasing NDG gene expression was observed in the three sample groups, including healthy controls (mean value 3.18), COVID-19 patients not on mechanical ventilation (Cov_noVent, mean value 5.11), and patients on mechanical ventilation (Cov_Vent, mean value 6.57). The *p*-values from multi-group Tukey’s test were 0 for Cov_noVent compared with controls, and 0 for Cov_Vent compared with Cov_noVent. Based on the four studies shown above, activation of neutrophil degranulation-related genes is highly correlated with oxygen requirement in COVID-19, which has been observed in whole blood and further validated in isolated neutrophils. 

### 3.4. Activation of Neutrophil Degranulation-Related Genes in Other Respiratory Diseases

COVID-19 is only one specific type of severe respiratory disease, while systemic hypoxia is a more common condition. Since systemic hypoxia is partially responsible for the activation of neutrophil degranulation-related genes in the blood of COVID-19 patients, it is expected that systemic hypoxia may also contribute to the activation of neutrophil degranulation-related genes in other types of severe respiratory diseases, including past and future pandemics. 

**Figure 3:**
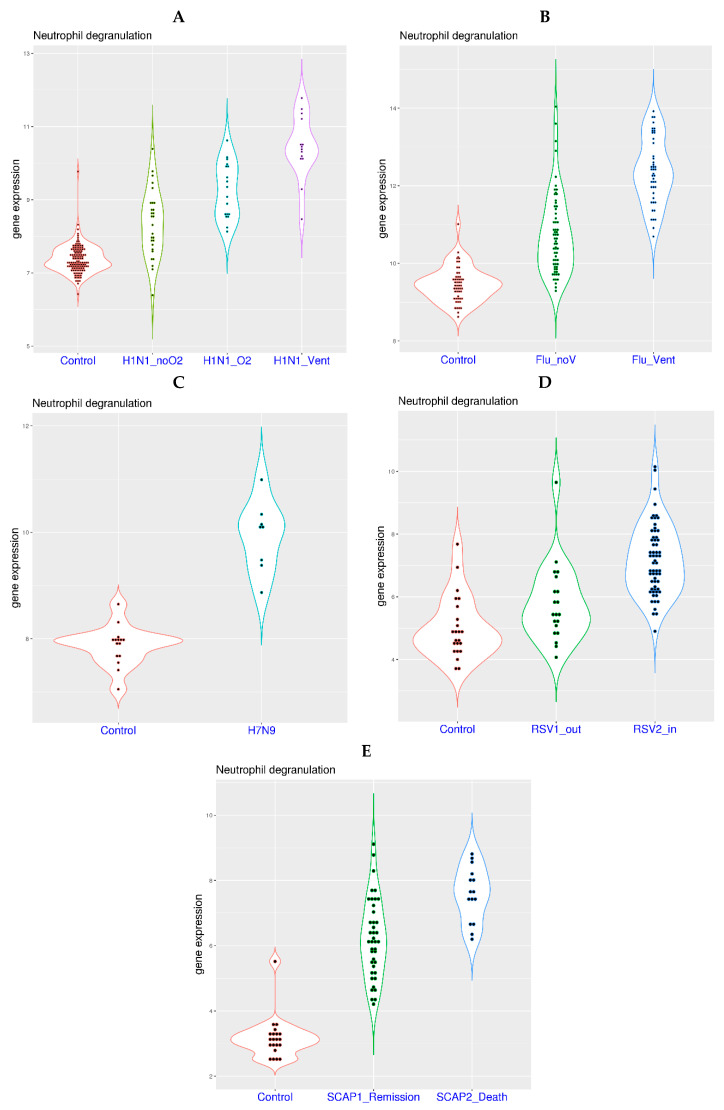
Activation of neutrophil degranulation-related genes in patients with other types of severe respiratory infection. **Upper left** (**A**): Activation of neutrophil degranulation-related genes in hospitalized H1N1 patients with room air (H1N1_noO2), oxygen supplementation (H1N1_O2) and mechanical ventilation (H1N1_Vent, dataset GSE111368). **Upper right** (**B**): Activation of neutrophil degranulation-related genes in hospitalized influenza A patients with or without the requirement of mechanical ventilation (Flu_Vent and Flu_noV, dataset GSE101702). **Middle left** (**C**): Activation of neutrophil degranulation-related genes in hospitalized H7N9 patients (dataset GSE114466). **Middle right** (**D**): Activation of neutrophil degranulation-related genes in patients with severe RSV infection (in-patients and out-patients, dataset GSE77087). **Lower panel** (**E**): Activation of neutrophil degranulation-related genes in patients with severe community acquired pneumonia (death or remission, dataset GSE196399).

H1N1 infection was responsible for the notorious Spanish flu in 1918. In a related study [30], similar correlation of systemic hypoxia and the activation of neutrophil degranulation-related genes in blood was observed (Figure 3A). More specifically, increasing NDG gene expression was observed in the four sample groups, including healthy controls (mean value 7.38), H1N1 patients on room air (H1N1_noO2, mean value 8.34), patients on supplementary oxygen (H1N1_O2, mean value 9.24), and patients on mechanical ventilation (H1N1_Vent, mean value 10.44). The *p*-values from multi-group Tukey’s test were 0 for H1N1_noO2 compared with controls, 3.4 × 10^−6^ for H1N1_O2 compared with H1N1_noO2, and 1.0 × 10^−7^ for H1N1_Vent compared with H1N1_O2.

Similarly, among patients with severe influenza A infection (Figure 3B) [32], an increased NDG gene expression was observed in the three sample groups, including the healthy controls (mean value 9.45), patients without ventilation requirement (Flu_noV, mean value 10.74), and patients requiring mechanical ventilation (Flu_Vent, mean value 12.37). The *p*-values from multi-group Tukey’s test were 0 for Flu_noV compared with controls, and 0 for Flu_Vent compared with Flu_noV. Based on these two studies, systemic hypoxia is also partially responsible for the activation of neutrophil degranulation-related genes in the past pandemic and severe seasonal flu.

In addition, activation of neutrophil degranulation-related genes has also been observed in other severe respiratory diseases. In a study on H7N9 infection with high fatality rate (Figure 3C) [31], H7N9 patients displayed significantly higher NDG expression (mean value 9.92) than healthy controls (mean value 7.87). The *p*-value from *t*-test was 1.37 × 10^−5^. In another study on respiratory syncytial virus (RSV) infection (Figure 3D), an increased NDG gene expression was observed in the three sample groups, including healthy controls (mean value 5.06), out-patients (RSV1_out, mean value 5.80), and in-patients (RSV2_in, mean value 7.19). The *p*-values from multi-group Tukey’s test were 0.07 for RSV1_out compared with controls, and 1.2 × 10^−5^ for RSV2_in compared with RSV1_out. In a third study on severe community acquired pneumonia (SCAP, Figure 3E) [34], non-surviving patients displayed significantly higher NDG expression (mean value 7.58) than surviving patients (mean value 6.22) and healthy controls (mean value 3.16). The *p*-values from multi-group Tukey’s test were 0 for surviving patients against healthy controls and 8.4 × 10^−5^ for non-surviving patients against surviving patients. Although information on oxygen requirement is not available for these three datasets, it is likely that systemic hypoxia is also partially responsible for the activation of neutrophil degranulation-related genes in H7N9 infection, RSV infection and SCAP.

### 3.5. Examination of Hypoxia Sensing Pathways in the Activation of Neutrophil Degranulation-Related Genes in Blood

**Figure 4:**
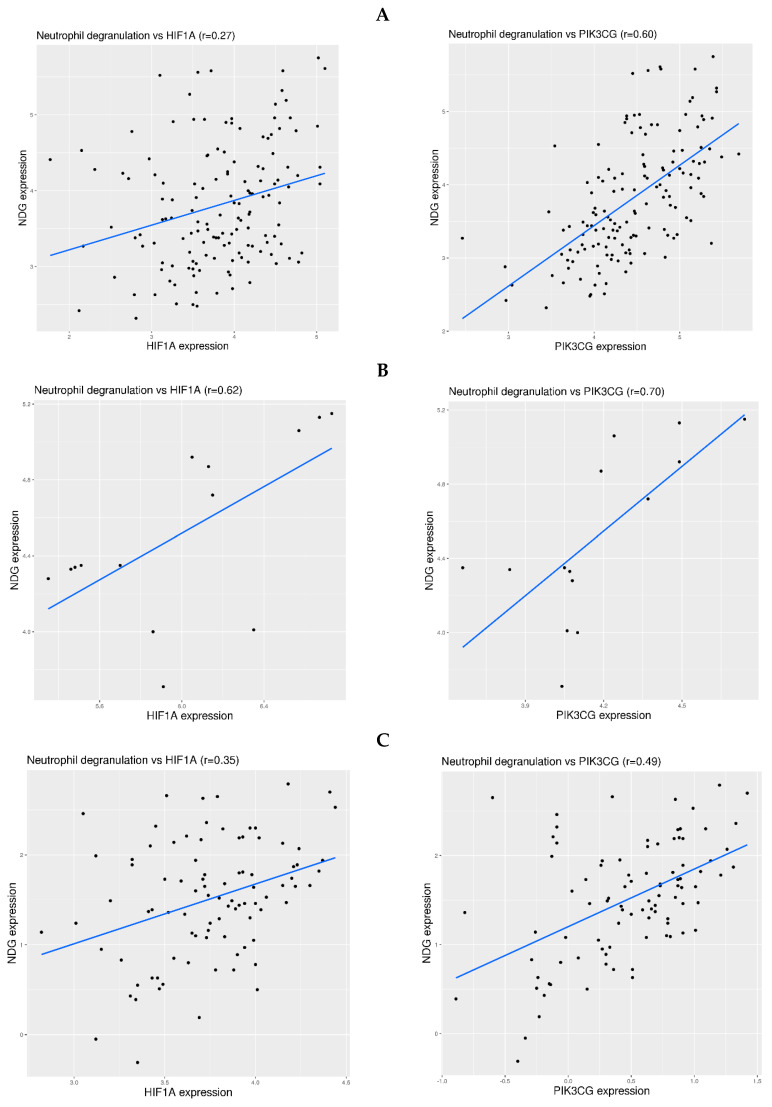

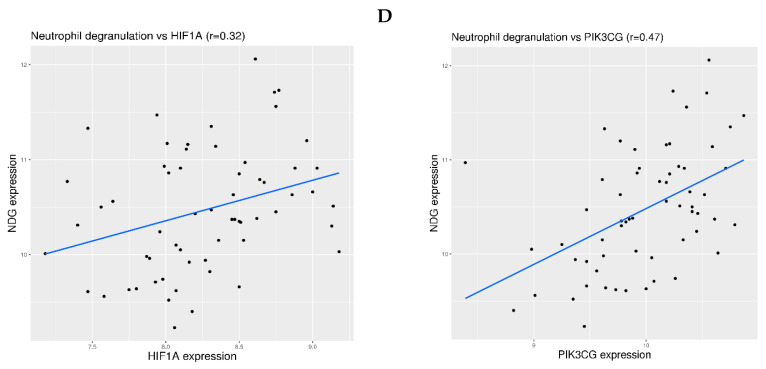
Correlation between HIF1A or PIK3CG expression and NDG expression in physiological and pathological hypoxia. **First row** (**A**): Correlation in the experiment on hypoxia induced by high altitude (dataset GSE196728). **Second row** (**B**): Correlation in the experiment on hypoxia induced by high intensity training at high altitude (dataset GSE164890). **Third row** (**C**): Correlation in the experiment on hypoxia induced by cardioembolic stroke (dataset GSE58294). **Fourth row** (**D**): Correlation in the experiment on hypoxia induced by ruptured intracranial aneurysm (dataset GSE36791). The blue lines indicate linear regression fitting of the data.

**Figure 5:**
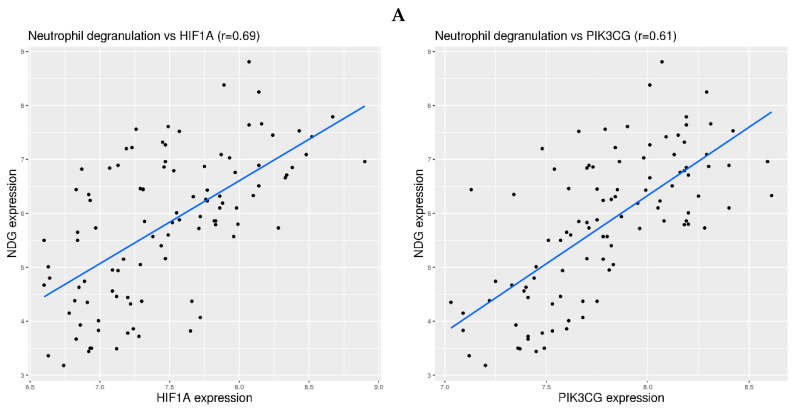

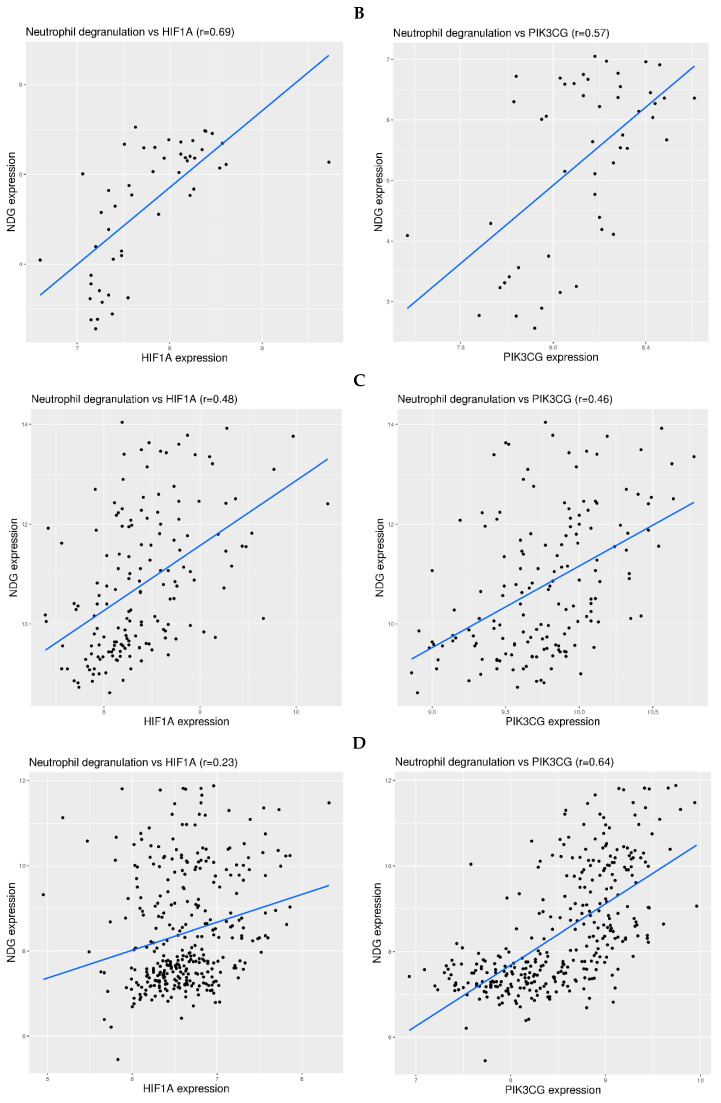
Correlation between HIF1A or PIK3CG expression and NDG expression in COVID-19 and other types of respiratory infections. **First row** (**A**): Correlation in an experiment on the whole blood of COVID-19 patients (dataset Zenodo 6120249). **Second row** (**B**): Correlation in an experiment on the isolated neutrophils of COVID-19 patients (dataset EGAS00001004503). **Third row** (**C**): Correlation in an experiment on the whole blood of H1N1 patients (dataset GSE111368). **Fourth row** (**D**): Correlation in an experiment on the whole blood of severe influenza A patients (dataset GSE101702). The blue lines indicate linear regression fitting of the data.

The mechanism for the regulation of gene expression can be very complicated. Nevertheless, it is still worth examining which hypoxia sensing pathway may be involved in the activation of neutrophil degranulation-related genes in blood. Two representative genes were examined, including HIF1A for HIF-dependent pathway and PIK3CG for HIF-independent pathway [35]. The correlation between HIF1A or PIK3CG expression and NDG expression was examined for all 13 datasets in Figure 1, Figure 2 and Figure 3. Overall, the correlation was more consistent in the infection-free hypoxia than that in the infection-related hypoxia. More specifically, moderate correlation (0.5 < r < 0.75) was observed between PIK3CG expression and NDG expression in altitude related-hypoxia (Figure 4A,B), where weak (0.25 < r < 0.5) or moderate correlation was observed between HIF1A expression and NDG expression. In comparison, weak correlation was observed in stroke-related hypoxia (Figure 4C,D). As for COVID-19, moderate correlation was observed in two datasets (Zenodo 6120249 for whole blood and EGAS00001004503 for isolated neutrophils) (Figure 5A,B). For other types of infection, weak correlation was observed in severe influenza A infection, and moderate correlation between PIK3CG and NDG expression was observed in H1N1 infection (Figure 5C,D). Thus, the mechanism for the regulation of neutrophil degranulation-related gene expression could be more complicated in severe respiratory infection.

## 4. Discussion

Hyper-inflammation is a common problem for severe COVID-19. To date, the most effective drugs for severe COVID-19 are immune modulators [2]. Immune dysregulation in COVID-19 starts early in the disease course and propagates during the process. One of the well-known immune features of COVID-19 is the muted interferon signaling [36]. However, targeting interferon signaling in COVID-19 has mixed results [37,38]. To gain more comprehensive understanding of immune dysregulation in COVID-19, investigations on blood transcriptome have been conducted by many independent groups. In the current study, consensus features from these studies have been revealed. The most prominent features were the activation of genes related to neutrophil degranulation and cell cycle. 

Compared to neutrophil degranulation, genes related to the cell cycle generally have a relatively low level of expression in peripheral blood, sometimes even non-detectable. Thus, many of the detected expression signals for cell cycle-related genes may be close to the noise level, casting doubt on the results. In contrast, genes related to neutrophil degranulation generally have medium to high level of expression in peripheral blood. In addition, 13 of the 16 most consensus genes were in the functional category of neutrophil degranulation, further highlighting the robustness of the signal. Therefore, neutrophil degranulation was chosen as the main focus of this work to represent immune dysregulation in COVID-19. Although a few interferon stimulated genes (ISGs) were also among the consensus genes, this functional category was not detected in the enrichment analysis. Thus, it was omitted in further examination. For the same reason, the down-regulated genes were also omitted in the mechanistic investigation.

For unbiased profiling and practical reasons, whole blood was the tissue source in most transcriptome studies on COVID-19. As mentioned above, the most consensus gene dysregulation in whole blood was found to be related to neutrophil degranulation. Ideally, it should be confirmed with similar protocols on isolated neutrophils. Therefore, we provide side-by-side comparison of neutrophil degranulation feature with whole blood and isolated neutrophils in Figure 2. The trend was identical in both whole blood and isolated neutrophils. Namely, gene expression related to neutrophil degranulation was higher in COVID-19 patients than that in healthy controls, and it was higher in patients with a higher level of oxygen requirement. Thus, activation of neutrophil degranulation-related genes in whole blood was confirmed with isolated neutrophils. In addition, the fold change was between 2.7 and 31.6 for the 13 most consensus NDG genes listed in Table 2. This could not be explained by the mere increase of neutrophil percentage in some patients, because neutrophil is already the most dominant blood cell type under normal conditions. Therefore, significant activation of neutrophil degranulation-related genes in blood is a fact for COVID-19. Activation of neutrophil degranulation-related genes has been reported in a few transcriptome studies in human patients and primate models [39,40]. In addition, the activation of neutrophil gene expression was shown to be correlated with oxygen requirement in a previous study [41].

In addition to transcriptome studies, activation of neutrophil degranulation has also been reported in proteomics studies. In a transcriptome study on isolated neutrophils (Figure 2C) [28], matched plasma proteomics measurement showed that many neutrophil degranulation markers were activated, including MPO, CD177, PADI4, ELANE, CTSG, LCN2, PRTN3, MMP8, MMP9, ARG1, and S100A12. Another plasma proteomics study also revealed the activation of several neutrophil degranulation markers [42], including MPO, LCN2, DEFA3, HP, S100A8, S100A9, and CRISP3. Another plasma proteomics study also identified the activation of several neutrophil degranulation markers [43], including PRTN3, LCN2, MMP8, ANXA3, and RETN. Activation of marker proteins LCN2, RETN, and MMP8 was also replicated in another plasma proteomics study [44]. In a proteomics study on nasopharyngeal swabs, activation of several markers was also found [45], including MPO, PRTN3, ELANE, CSTG, AZU1, and TCN1. Interestingly, the corresponding genes of the above-listed marker proteins for neutrophil degranulation were all among our list of 263 consensus up-regulated genes.

The current work confirmed the activation of neutrophil degranulation-related genes in COVID-19. However, more importantly, it is critical to find potential upstream causal factors, especially those with practical ways of clinical intervention. Towards this end, it was found that systemic hypoxia by itself—be it physiological or pathological—can lead to activation of neutrophil degranulation-related genes in blood, and the latter was highly correlated with oxygen deprivation in COVID-19 patients. Therefore, activation of neutrophil degranulation-related genes in blood could be partially attributed to systemic hypoxia in COVID-19. Unlike those in milder respiratory diseases such as seasonal flu, systemic hypoxia has been widely reported in COVID-19 [7]. Systemic hypoxia may originate from damage to the lungs or circulation. Either way, early intervention can alleviate symptoms and may prevent harmful downstream hyper-inflammation. It has been reported that unnoticed hypoxia may lead to poor outcomes in COVID-19 patients [8]. Therefore, close monitoring of systemic hypoxia upon symptom onset can be an effective strategy to help prevent the development of severe COVID-19.

It should be noted that factors other than systemic hypoxia may also contribute to the activation of neutrophil degranulation-related genes in COVID-19. Based on data from Figure 2A,C, it was evident that some COVID-19 patients without the requirement of supplementary oxygen also displayed activation of neutrophil degranulation-related genes. It is still yet to be found what the mechanism may be. The mechanism could be similar to host response to bacterial infection [46,47,48]—however, it may also be different. Regardless, considering that gene expression related to neutrophil degranulation is highly correlated with disease severity in COVID-19, it deserves further in-depth investigation in future studies. 

As the end of the COVID-19 pandemic is approaching, it is time to prepare for the next pandemic. What we have learned from this pandemic will be valuable assets for the future. We have shown that systemic hypoxia has contributed to severe disease for COVID-19 and some other respiratory diseases, including H1N1 infection, H7N9 infection, severe influenza A infection, severe RSV, and severe community-acquired pneumonia. Therefore, it is advisable that close monitoring of systemic hypoxia becomes a common practice for the handling of severe respiratory diseases including future pandemics.

Investigation of gene dysregulation in COVID-19 has also been conducted on tissues other than blood [49,50,51]. It seems logical to compare the findings from blood and other tissues. However, the main finding in this work was the activation of neutrophil degranulation-related genes in the blood of COVID-19 patients. Gene expression of neutrophil degranulation signature is likely restricted to neutrophils. Nevertheless, the signature gene panel was examined in a few transcriptome datasets related to lung and nasopharyngeal swab samples, and the expression of many genes could not be detected. 

The main limitation of this work lies in the experimental design of available transcriptome datasets. Although the causal link between hypoxia and neutrophil degranulation has been reported in certain conditions [35], the mechanism may not be applicable to COVID-19 and other severe respiratory infections. It is more straightforward to comprehend the causal link between hypoxia and activation of neutrophil degranulation-related genes in blood in the two altitude-related studies because the measurement was conducted on the same group of healthy volunteers. For the studies on COVID-19 or other types of infection, the cross-sectional nature of the study design will make the causal link much more complex. It would be ideal to conduct the measurements on the same group of healthy volunteers using experimental challenging of a specific virus. However, it would be very difficult to execute considering the risk. 

As for the molecular link between hypoxia and neutrophil degranulation in blood, we examined HIF-dependent and HIF-independent pathways using the correlation between two representative genes and NDG expression. It seems that the correlation was more consistent in altitude-related hypoxia than that in COVID-19 or other respiratory infections. It shall be noted that the regulation of gene expression is very complex, involving protein and epigenetic regulation that may not be reflected at the gene expression level. However, this complexity is beyond the scope of the current work.

## 5. Conclusions

Based on over a dozen of transcriptome datasets, we revealed a set of consensus genes dysregulated in the blood of COVID-19 patients. These consensus genes were mainly enriched in neutrophil degranulation and cell cycle, the former being the most consensus functional category. Furthermore, the potential upstream causal factors were investigated. Systemic hypoxia was found to be able to activate neutrophil degranulation-related genes in blood. In addition, oxygen requirement was highly correlated with the activation of neutrophil degranulation-related genes in COVID-19 patients in both whole blood and isolated neutrophils. Therefore, activation of neutrophil degranulation-related genes in COVID-19 could be partially attributed to systemic hypoxia. As for the molecular mechanism, a moderate correlation was observed between HIF1A or PIK3CG and NDG expression in two of the COVID-19 datasets. It is therefore advisable to have continuous and convenient monitoring of systemic hypoxia in COVID-19 patients as early as possible. This strategy may also be applicable to future pandemics and other severe respiratory diseases.

## Figures and Tables

**Table 1:** COVID-19 transcriptome datasets for consensus gene dysregulation in blood.

Accession ID	Sources	Case Number	Control Number	Reference
GSE152641	Whole blood	62	24	[9]
GSE161731	Whole blood	77	19	[10]
GSE161777	Whole blood	11	14	[11]
GSE171110	Whole blood	44	10	[12]
GSE179850	Whole blood	31	16	[13]
GSE185557	Whole blood	11	10	[14]
GSE189990	Whole blood	20	4	[15]
GSE196822	Whole blood	40	9	[16]
GSE213313	Whole blood	83	11	[17]
GSE217948	Whole blood	395	72	[18]
GSE223885	Whole blood	10	10	[19]
Zenodo 6120249	Whole blood	78	10	[20]
HRA000238	Whole blood	12	4	[21]

**Table 2:** Activation of genes related to neutrophil degranulation in COVID-19 patients.

Gene Symbol	Frequency (logFC > 1)	Median logFC	Correlation w/Severity
ARG1	13	2.87	0.68
CEACAM8	13	2.93	0.67
RNASE2	13	2.01	0.77
CD177	12	4.98	0.61
CKAP4	12	1.43	0.70
ELANE	12	2.92	0.65
HP	12	3.13	0.75
MCEMP1	12	2.44	0.69
MMP9	12	2.9	0.67
MPO	12	2.27	0.70
S100A12	12	2.02	0.69
S100A9	12	1.63	0.72
TCN1	12	2.18	0.69

Note: These 13 genes were upregulated >2 fold in at least 12 of the 13 datasets on the whole blood of COVID-19 patients. The correlation with disease severity was obtained from dataset GSE217948.

**Table 3:** Transcriptome datasets for the examination of the link between hypoxia and gene expression related to neutrophil degranulation in blood.

Accession ID	Sources	Condition	Sample Numbers	Ref
GSE164890	Whole blood	Training at high altitude	7 athletes Before and after training	[23]
GSE196728	Whole blood	High altitude	8 subjects, 3 altitudes, 6 time points	[24]
GSE36791	Whole blood	Stroke	18 controls, 43 patients	[25]
GSE58294	Whole blood	Stroke	23 controls, 23 patients, 3 time points	[26]
Zenodo 6120249	Whole blood	COVID-19	10 controls, 91 patients	[20]
GSE157103	Whole blood	COVID-19	50 ICU patients, 50 non-ICU patients	[27]
GSE212041	Neutrophils	COVID-19	8 controls, 299 patients	[28]
EGAS00001004503	Neutrophils	COVID-19	10 controls, 39 patients	[29]
GSE111368	Whole blood	H1N1	130 controls, 198 patients	[30]
GSE114466	Whole blood	H7N9	15 controls, 8 patients	[31]
GSE101702	Whole blood	Influenza A	52 controls, 107 patients	[32]
GSE77087	Whole blood	RSV	23 controls, 81 patients	[33]
GSE196399	Whole blood	SCAP	21 controls, 56 patients	[34]

## Data Availability

All the datasets analyzed in the current work are publicly available. Most of the transcriptome datasets are available at GEO (gene expression omnibus, https://www.ncbi.nlm.nih.gov/geo/, accessed on 15 May 2023). For more details, please refer to the Methods section.

## Supplementary Materials

The following supporting information can be downloaded at: https://www.mdpi.com/article/10.3390/v16020201/s1, Table S1: Consensus DEGs in the blood of COVID-19 patients; Table S2: Top 20 pathways from the ToppGene functional enrichment analysis.
