# Supplementary material for: Hypoxia and Activation of Neutrophil Degranulation-Related Genes in the Peripheral Blood of COVID-19 Patients
Source: Viruses. 2024 Jan 28;16(2):201. doi: 10.3390/v16020201 (PMC10891603; doi:10.3390/v16020201)
Supplement: Supplementary file 1 [file viruses-16-00201-s001.zip › Supplementary Table S2.pdf]

Supplementary Table S2 Top 20 pathways with significant enrichment from ToppGene analysis.

**Pathway**

| | ID | Name | Source | pValue | FDR B&H | FDR B&Y | Bonferroni | Genes from Input | Genes in Annotation |
|---|---|---|---|---|---|---|---|---|---|
| 1 | M27620 | REACTOME NEUTROPHIL DEGRANULATION | Reactome Pathways | 2.112E-35 | 3.964E-32 | 3.217E-31 | 3.964E-32 | 60 | 478 |
| 2 | MM15330 | REACTOME NEUTROPHIL DEGRANULATION | Reactome Pathways | 2.699E-30 | 2.533E-27 | 2.056E-26 | 5.066E-27 | 57 | 522 |
| 3 | M5336 | REACTOME CELL CYCLE MITOTIC | Reactome Pathways | 2.654E-13 | 1.660E-10 | 1.347E-9 | 4.981E-10 | 38 | 560 |
| 4 | M543 | REACTOME CELL CYCLE | Reactome Pathways | 5.368E-13 | 2.382E-10 | 1.933E-9 | 1.008E-9 | 42 | 692 |
| 5 | MM15332 | REACTOME ANTIMICROBIAL PEPTIDES | Reactome Pathways | 6.345E-13 | 2.382E-10 | 1.933E-9 | 1.191E-9 | 15 | 70 |
| 6 | M39650 | WP CELL CYCLE | WikiPathways | 1.916E-12 | 5.995E-10 | 4.865E-9 | 3.597E-9 | 18 | 120 |
| 7 | M7963 | KEGG CELL CYCLE | KEGG Legacy Pathways | 3.919E-12 | 1.051E-9 | 8.528E-9 | 7.356E-9 | 18 | 125 |
| 8 | M16647 | REACTOME CELL CYCLE CHECKPOINTS | Reactome Pathways | 4.997E-12 | 1.172E-9 | 9.514E-9 | 9.380E-9 | 26 | 291 |
| 9 | MM15388 | REACTOME CELL CYCLE CHECKPOINTS | Reactome Pathways | 3.169E-10 | 6.609E-8 | 5.363E-7 | 5.948E-7 | 23 | 274 |
| 10 | M1040 | REACTOME G1 S SPECIFIC TRANSCRIPTION | Reactome Pathways | 8.037E-10 | 1.508E-7 | 1.224E-6 | 1.508E-6 | 9 | 29 |
| 11 | M27627 | REACTOME ANTIMICROBIAL PEPTIDES | Reactome Pathways | 1.147E-9 | 1.958E-7 | 1.589E-6 | 2.154E-6 | 14 | 98 |
| 12 | MM14635 | REACTOME CELL CYCLE | Reactome Pathways | 2.756E-9 | 4.311E-7 | 3.498E-6 | 5.173E-6 | 33 | 600 |
| 13 | M39678 | WP RETINOBLASTOMA GENE IN CANCER | WikiPathways | 3.453E-9 | 4.985E-7 | 4.045E-6 | 6.481E-6 | 13 | 89 |

| | | | | | | | | | |
|---|---|---|---|---|---|---|---|---|---|
| 14 | M26971 | REACTOME POLO LIKE KINASE MEDIATED EVENTS | Reactome Pathways | 3.946E-9 | 5.290E-7 | 4.293E-6 | 7.406E-6 | 7 | 16 |
| 15 | M808 | REACTOME CYCLIN A B1 B2 ASSOCIATED EVENTS DURING G2 M TRANSITION | Reactome Pathways | 5.498E-9 | 6.450E-7 | 5.234E-6 | 1.032E-5 | 8 | 25 |
| 16 | MM15379 | REACTOME CYCLIN A B1 B2 ASSOCIATED EVENTS DURING G2 M TRANSITION | Reactome Pathways | 5.498E-9 | 6.450E-7 | 5.234E-6 | 1.032E-5 | 8 | 25 |
| 17 | M176 | PID FOXM1 PATHWAY | PID Pathways | 1.862E-8 | 2.056E-6 | 1.669E-5 | 3.496E-5 | 9 | 40 |
| 18 | M19381 | REACTOME G2 M CHECKPOINTS | Reactome Pathways | 1.772E-7 | 1.848E-5 | 1.500E-4 | 3.326E-4 | 15 | 167 |
| 19 | M532 | REACTOME G0 AND EARLY G1 | Reactome Pathways | 2.608E-7 | 2.577E-5 | 2.091E-4 | 4.896E-4 | 7 | 27 |
| 20 | M129 | PID PLK1 PATHWAY | PID Pathways | 9.728E-7 | 9.130E-5 | 7.409E-4 | 1.826E-3 | 8 | 46 |
